# Cuproptosis-Related Signature Predicts the Prognosis, Tumor Microenvironment, and Drug Sensitivity of Hepatocellular Carcinoma

**DOI:** 10.1155/2022/3393027

**Published:** 2022-11-16

**Authors:** Xiangjun Qi, Jiayun Guo, Guoming Chen, Caishan Fang, Leihao Hu, Jing Li, Chi Zhang

**Affiliations:** ^1^The First Clinical School of Guangzhou University of Chinese Medicine, Guangzhou, China; ^2^School of Chinese Medicine, Li Ka Shing Faculty of Medicine, The University of Hong Kong, Hong Kong S.A.R, China; ^3^The First Hospital of Hunan University of Chinese Medicine, Changsha, China; ^4^The Second Clinical School of Beijing University of Chinese Medicine, Beijing, China

## Abstract

**Background:**

Copper (Cu) metabolism is strongly associated with liver disease. Cuproptosis is a novel format of cell death, and cuproptosis-related genes (CRGs) were identified. However, the role of CRGs in Hepatocellular Carcinoma (HCC) remains unknown.

**Method:**

The mRNA transcriptome profiling data, somatic mutation data, and copy number gene level data of The Cancer Genome Atlas-Liver Hepatocellular Carcinoma project (TCGA-LIHC) were downloaded for subsequent analysis. Molecular characterization analysis of CRGs, including differential gene expression analysis, mutation analysis, copy number variation (CNV) analysis, Kaplan-Meier analysis, and immune regulator prioritization analysis, was implemented. The nonnegative matrix factorization (NMF) approach was used to identify the CRG-related molecular subtypes. Principal component analysis was adopted to verify the robustness and reliability of the molecular subtype. The least absolute shrinkage and selection operator regression analysis was performed to construct the prognostic signature based on differentially expressed genes between molecular subtypes. The survival characteristics of the molecular subtype and the signature were analyzed. The Gene Set Variation Analysis was performed for functional annotation. The immune landscape analysis, including immune checkpoint gene analysis, single sample gene set enrichment analysis, tumor immune dysfunction and exclusion (TIDE) analysis, immune infiltration cell, and tumor mutation burden analysis (TMB), was conducted. The ability of the signature to predict conventional anti-HCC agent responses was evaluated. The signature was validated in the LIRI-JP cohort and the IMvigor210 cohort.

**Result:**

A total of 13 CRGs are differentially expressed between the tumor and normal samples, while the mutation of CRGs in HCC is infrequent. The expression of CRGs is associated with the CNV level. Fourteen CRGs are associated with the prognosis of HCC. Two clusters were identified and HCC patients were divided into 2 groups with a cutoff risk score value of 1.570. HCC patients in the C1 cluster and high-risk have a worse prognosis. The area under the receiver operating characteristic curve for predicting 1-, 2-, and 3-year overall survival is 0.775, 0.768, and 0.757 in the TCGA-LIHC cohort, and 0.811, 0.741, and 0.775 in the LIRI-JP cohort. Multivariate Cox regression analysis indicates that the signature is an independent prognostic factor. Pathways involved in metabolism and gene stability and immune infiltration cells are significantly enriched. Immune checkpoint genes are highly expressed in the C1 cluster. TMB is positively correlated with the risk score. HCC patients in the high-risk group are more likely to benefit from conventional anti-HCC agents and immune checkpoint inhibitor therapies.

**Conclusion:**

The molecular characterization of CRGs in HCC is presented in this study, and a successful prognostic signature for HCC based on the cuproptosis-related molecular subtype was constructed.

## 1. Introduction

Hepatocellular Carcinoma (HCC), which accounts for 75-85% of primary liver cancer cases, remains a major public health issue worldwide [1]. It is reported in the Global Cancer Statistics 2020 that the newly diagnosed cases and death cases of primary liver cancer are sorted by sixth and third, respectively. The 5-year survival rates in China and the United States are only 12.1% [2] and 18% [3]. HCC patients at an early stage can be cured by surgical interventions, whereas limited drugs are effective in advanced patients. Few molecular targeted drugs have been successfully applied to HCC except for several multikinase inhibitors and antiangiogenesis drugs, such as sorafenib and bevacizumab. What is more, sketchy molecular testing for directing HCC treatment has been conducted [4]. Immune checkpoint inhibitor (ICI) therapies have shown encouraging efficacy in advanced HCC patients. Atezolizumab combined with bevacizumab prolonged the median progression-free survival to 6.8 months [5]. Not only is ICI therapy facing resistant factors originating from the expression level of immune checkpoint point genes, tumor immune microenvironment, and tumor mutation burden (TMB) but also the specific molecular mechanisms remain unclear [6].

HCC is characterized by high heterogeneity, which influences tumor progression, aggression, and response to antitumor agents [7]. Gene changes such as somatic mutations, epigenetic variations, and large-scale genomic alterations contribute to the occurrence of heterogeneity and result in different molecular subtypes [8]. Differential expression of a heterogeneous genome brings out the discrepant biological behavior of tumor cells and their tumor microenvironment. Thus, RNA sequencing is a potent approach used to identify molecular subtypes. A number of studies have proved that gene expression profile-based signatures could predict the prognosis [9] and recurrence of HCC [10] as well as classify the tumor microenvironment [11].

Copper (Cu) metabolism, which has been identified to be associated with liver diseases, such as Wilson disease [12], liver cirrhosis [13], and HCC [14], is recently identified to be involved in cell death. Cuproptosis is a brand-new form of regulated cell death brought forward in 2022 [15]. It is distinct from all other known types of cell death, including apoptosis, ferroptosis, pyroptosis, and necroptosis. A series of cuproptosis-related genes (CRGs) were recognized through a genome-wide CRISPR/Cas9 screen by researcher [15]. Previous studies have demonstrated the prognostic value and molecular subtype of ferroptosis-related genes [16] and pyroptosis-related genes [17] in HCC. However, little is known about cuproptosis in this regard.

In this study, we recognized the cuproptosis-related molecular subtype of HCC and developed a cuproptosis-related prognostic signature.

## 2. Materials and Methods

### 2.1. Data Preparation

The mRNA transcriptome profiling of The Cancer Genome Atlas-Liver Hepatocellular Carcinoma (TCGA-LIHC) project with transcripts per million format was downloaded and further transformed by log2 (*x* + 1). The mRNA transcriptome profiling of the LIRI-JP project with fragments per kilobase of exon per million fragments format was obtained from the International Cancer Genome Consortium website (ICGC, https://dcc.icgc.org/) [18], and subsequently transformed into transcripts per million format and normalized by log2 (*x* + 1). Somatic mutation data with VarScan2 variant aggregation and masking format and copy number gene level data of the TCGA-LIHC project were obtained from the UCSC Xena website (https://xena.ucsc.edu/) [19]. The clinical data of the TCGA-LIHC project and the LIRI-JP project were downloaded from the TCGA website (https://portal.gdc.cancer.gov/) and the ICGC website, respectively. The mRNA transcriptome profiling and clinical data of the IMvigor210 study [20] were downloaded with the R IMvigor210CoreBiologies package. The clinical samples lacking survival state or overall survival (OS) were excluded. A gene list of CRGs was extracted from the research conducted by Tsvetkov et al., including 10 genes identified by whole genome CRISPR/Cas9 screen analysis, 3 Cu transporter-related genes, and 3 tricarboxylic acid cycle-related genes [15].

### 2.2. Molecular Characterization of CRGs in HCC

#### 2.2.1. Differential Expression Analysis of CRGs

To identify the expression differences of CRGs between the normal and tumor samples, the R limma and ggpubr package were used to generate the boxplots for the comparison.

#### 2.2.2. Mutation Analysis of CRGs

The mutation frequency and mutation type of CRGs were visualized by the R maftools package.

#### 2.2.3. Copy Number Variation (CNV) Analysis of CRGs

The occurrence of CNV was presented in a barplot, and the distribution of CRGs in chromosomes was depicted by a circle plot with the R RCircos package. The CNV data was divided into three levels, including single deletion, normal, and single gain, and the correlations between these three categorical variables (CNV data and mRNA expression data) were tested.

#### 2.2.4. Kaplan-Meier Analysis and Correlation Analysis of CRGs

To specify the prognostic value of single CRG in HCC, R limma, survival, and survminer packages were used for drawing the Kaplan-Meier (K-M) curve and statistical test. The correlations between expression levels of CRGs were calculated and visualized by the R igraph package.

### 2.3. Construction and Validation of Cuproptosis-Related Molecular Subgroup and Signature

#### 2.3.1. Immune Regulator Prioritization Analysis of CRG Set

The CRG set prioritization module includes T cell dysfunction, T cell exclusion score, Cox proportional-hazards (Cox-PH) regression score in the immune checkpoint inhibitor (ICB) cohort, and log-fold change (logFC) in CRISPR screens was established with the usage of the Tumor Immune Dysfunction and Exclusion (TIDE) website (http://tide.dfci.harvard.edu/). The T dysfunction score and T cell exclusion score indicate the interactions between gene expression level and cytotoxic T cells as well as gene expression level and immunosuppressive cells [21]. The Cox-PH score was associated with ICB therapy response, and the logFC score was employed to sift genes that can interact with lymphocyte-mediated tumor killing [21].

#### 2.3.2. Construction of Cuproptosis-Related Molecular Subtype

The nonnegative matrix factorization (NMF) approach, an unsupervised clustering analysis method, was performed to identify the presence of underlying molecular subgroups associated with cuproptosis. The R NMF package was employed in this process, and the detailed method used in NMF-clustering analysis was “brunet.” The consensus matrix heat map and cophenetic correlation coefficient were used to determine the cluster number. Principal component analysis (PCA) was adopted to verify the robustness and reliability of the molecular subtypes.

#### 2.3.3. Construction of Cuproptosis-Related Signature

Differential gene expression analysis was initially conducted for the molecular subgroups obtained from the NMF-clustering analysis. Differentially expressed genes (DEGs) among subtypes were screened with a threshold value ∣logFC | = 0.585 and adjusted *p* value =0.05. Univariate Cox analysis was performed on DEGs and DEGs with a *p* value <0.05 were retained for subsequent analysis. The least absolute shrinkage and selection operator (LASSO) regression analysis with a repeated time of 1000 was used to improve the model stability. Multivariate Cox analysis was performed based on DEGs after LASSO regression analysis. Finally, a risk score for each patient was calculated using the following formula:

(1) $$\begin{array}{c} \text{RiskScore}=\overset{n}{\underset{i=1}{\sum }}\beta iSi. \end{array}$$

The “*βi*” and the “*Si*” in the formula represent the coefficient and expression level of genes, respectively. Patients were divided into high- and low-risk groups according to a cutoff point generated by the Akaike information criterion (AIC). R survival, survminer, and glmnet packages were used in this section.

#### 2.3.4. Correlations between Molecular Subtype and Signature

The differences in risk scores between the molecular subtypes were analyzed with a statistical test using the R ggpubr package. A Sankey diagram was plotted to show the distribution of patients by molecular subtype, risk score, and survival status.

#### 2.3.5. Survival and Clinical Characteristics of Molecular Subtype and Signature

A K-M analysis was conducted to compare the survival curve between the molecular subtype and the risk score group. The differences in the clinical phenotype, including age, gender, histologic grade, and TNM stage, were measured as well. Receiver Operating Characteristic (ROC) curves were used to test the diagnostic performance of the signature. The ROC curves for 1-, 2-, and 3-year were plotted and further compared with the clinical ROC curve. Univariate and multivariate Cox regression analyses were adopted to determine whether the signature could be regarded as an independent prognostic factor. R packages including survival, pheatmap, pbapply, survivalROC, survminer, glmnet, and ggupbr were used for these operations.

#### 2.3.6. Differential Gene Expression Analysis of CRGs

In order to recognize the different expression of CRGs between the molecular subtypes and the risk groups, we used the R limma and the ggpubr package to generate the boxplots for the comparison.

#### 2.3.7. Gene Set Variation Analysis (GSVA) and Functional Enrichment

GSVA analysis was conducted to identify the biological pathway differences between the molecular subtypes and the risk groups. The KEGG subset file with gene symbol format was downloaded from the MSigDB database (https://www.gsea-msigdb.org/gsea/msigdb/). The threshold values used to screen significantly differentially enriched pathways were set as follows: adjusted *p* value =0.05. R limma, GSEABase, GSVA, and pheatmap package were utilized.

#### 2.3.8. Immune Landscape of Molecular Subtype and Signature

The HCC-related immune checkpoint genes were extracted from Harkus et al.'s study, [6] and the expression levels of them were compared in accordance with diverse molecular subtypes and risk groups. The single sample gene set enrichment analysis (ssGSEA) algorithm was used to evaluate the immune cells and immune functions in tumor immune microenvironment. The infiltration abundance level of 23 types of immune cells and enrichment level of 13 types of immune functions was calculated and compared between the groups. The TIDE score, including dysfunction, exclusion, integrated TIDE score, and microsatellite instability (MSI) score of each HCC sample, were computed on the TIDE website. To evaluate the ratio of immune-stromal components in the tumor microenvironment, the R estimate package was utilized to calculate the StromalScore, ImmuneScore, and ESTIMATEScore. The immune scores were further compared between the groups. Moreover, the correlation between risk score and tumor-infiltrating immune cells, whose infiltration level was estimated by 7 algorithms including TIMER [22], quanTIseq [23], EPiC [24], CIBERSORT [25], xCell [26], MCP-counter [27], and CIBERSORT-ABS [28], was tested. The correlation between risk score and tumor mutation burden (TMB) was also tested.

#### 2.3.9. Anti-HCC Agent Response Prediction

Gene expression and drug sensitivity data used for drug response prediction were downloaded from the Genomics of Drug Sensitivity in Cancer website (GDSC, https://www.cancerrxgene.org). The R package pRRophetic was utilized to calculate the half-maximal inhibitory concentration (IC_50_) of 4 conventional anti-HCC agents, including sorafenib, cisplatin, paclitaxel, and gemcitabine, for each TCGA-LIHC project sample. The difference in IC_50_ between high- and low-risk groups was compared.

#### 2.3.10. Validation of the Constructed Signature

The LIRI-JP cohort was used to validate the constructed signature. A K-M analysis was conducted to compare the survival between the low-risk and the high-risk patients in the LIRI-JP cohort. A ROC diagram was plotted, and a corresponding area under curve (AUC) value was calculated to evaluate the accuracy of the signature. Univariate and multivariate Cox regression analyses were adopted to determine whether the signature could be regarded as an independent prognostic factor in the LIRI-JP cohort. The ability of the signature to predict ICIs' response was evaluated in the IMvigor210 cohort. The distribution of risk scores and risk status between complete response (CR)/partial response (PR) and stable disease (SD)/progressive disease (PD) was compared.

#### 2.3.11. Statistical Analysis

All statistical operators were conducted with R 4.0.3 software. A log-rank test was used to compare the differences between the survival curves generated by K-M analysis. The comparison and correlation analysis in continuous data were the Wilcox test and the Spearman test. Differences in categorical data were compared using *χ*^2^ or the Kruskal test. A *p* value below 0.05 was considered a statistically significant difference.

## 3. Results

**Figure 1:**
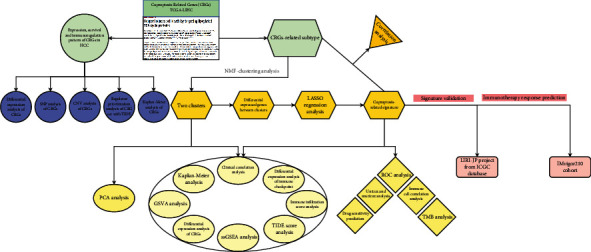
Overall design of the study.

A total of 424 mRNA transcriptome samples, which consist of 374 cancer and 50 normal samples, were obtained from the TCGA-LIHC project, as well as 371 clinical samples were retained after filtration. There were 231 HCC samples enrolled from the ICGC database and 381 ICI-treated tumor samples enrolled from the IMvigor210 cohort for validation.  Figure 1 presents the overall design of the study.

### 3.1. Identification of Differentially Expressed, Prognosis-Related, and Immune-Related CRGs in HCC

**Figure 2:**
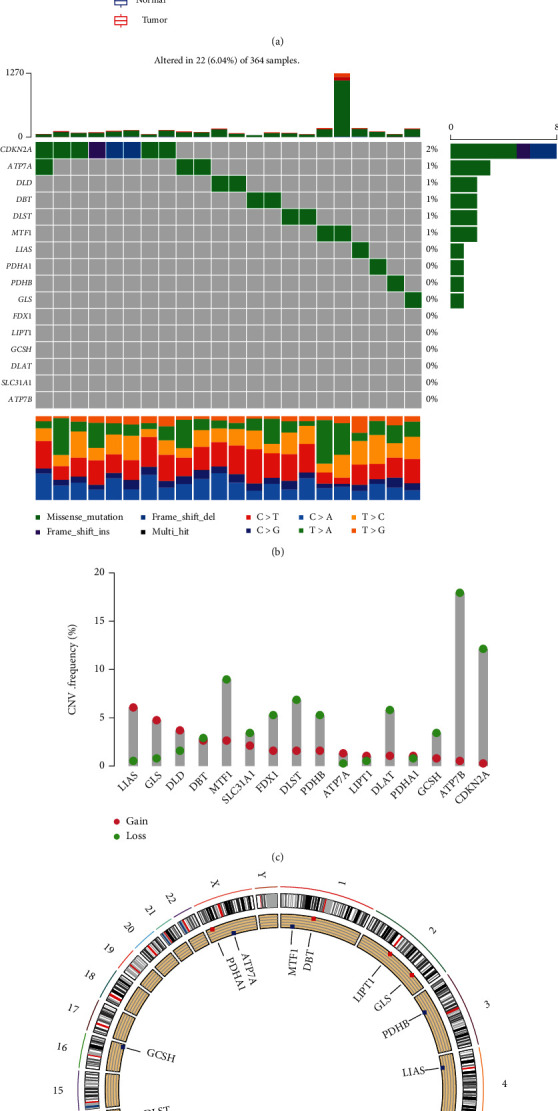
Molecular characterization of CRGs in HCC. (a) Differential expression of CRGs between normal and tumor samples. (b) Waterfall map of CRG mutation. (c) CNA frequency of CRGs. (d) Circos plot of detected CNVs in CRGs. ^∗∗∗^represents *p* < 0.001.

**Figure 3:**
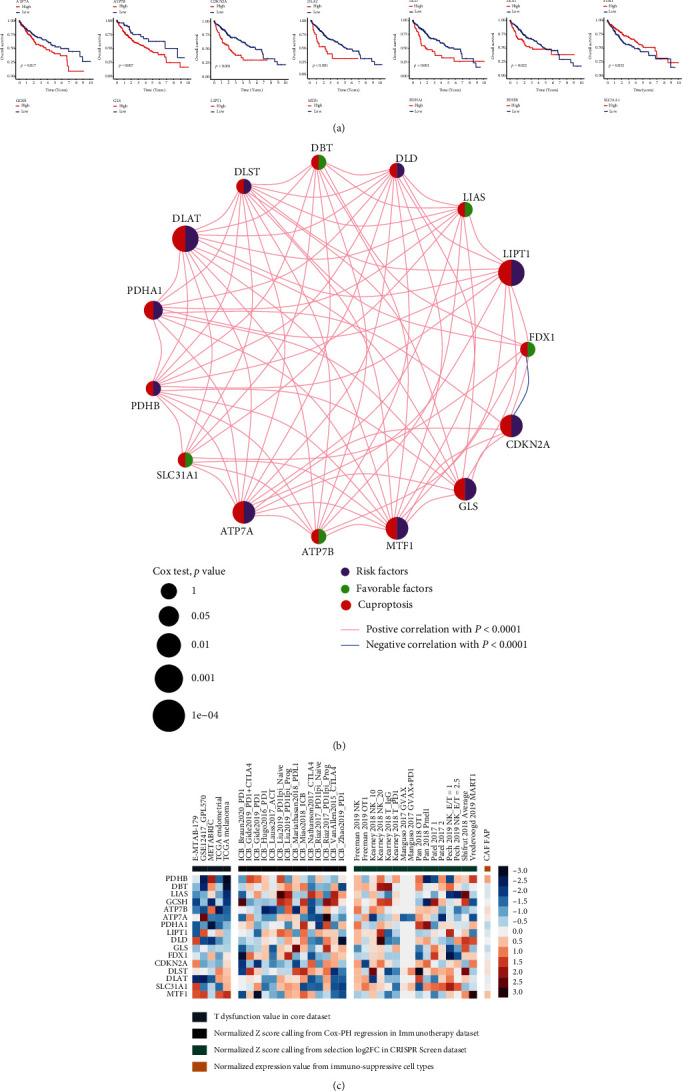
Survival and immune characterization of CRGs in HCC. (a) The K-M plots of CRGs associated with prognosis. (b) Correlation network of CRGs. (c) TIDE analysis of CRG set.

A total of 16 CRGs were obtained from Tsvetkov et al.'s study, and their molecular characteristics are presented in Figure 2. The expression level of DBT and SLC31A1 in normal samples is significantly higher than in tumor samples, while LIPT1, LIAS, DLD, DLST, DLAT, PDHA1, PDHB, ATP7A, MTF1, GLS, and CDKN2A are the opposite (Figure 2(a)). The incidence of mutation in CRGs in HCC is 6.04% and is dominated by missense mutations (Figure 2(b)). CDKN2A is the most mutated gene among CGRs. ATP7A and CDKN2A, which are located on chromosomes X and 6, respectively, harbor a high frequency of CNV-deletion (Figures 2(c) and 2(d)). Supplementary Figure 1 indicates that the occurrence of CNV in DLAT, CDKN2A, PDHB, DLST, ATP7B, GCSH, MTF1, LIAS, FDX1, DBT, DLD, and GLS is significantly associated with gene expression levels. The K-M analysis of single CRG demonstrates that the overall survival of HCC patients in high expression level of ATP7B, FDX1, and SLC31A1 are superior to those in the low expression group (Figure 3(a)). High expression levels of ATP7A, CDKN2A, DLAT, DLD, DLST, GCSH, GLS, LIPT1, MTF1, PDHA1, and PDHB are the risk factors for HCC patients (Figures 3(a) and 3(b)). TIDE analysis of CRG set indicates that the low expression levels of GCSH and PDHA1 is associated with the T cell dysfunction in all datasets catalogued (Figure 3(c)), while the high expression levels of GCSH and PDHA1 are associated with the response of immune checkpoint inhibitors in Miao et al. [29] and Rizvi et al. [30]. The majority of CRGs are differentially expressed between normal and tumor samples and are associated with the prognosis of HCC, which suggests that CRGs are of importance in the development and progression of HCC.

### 3.2. Construction and Validation of CRG-Related Molecular Subtype

#### 3.2.1. Differentially Expressed CRGs and Survival Differences between the Molecular Subtypes

**Figure 4:**
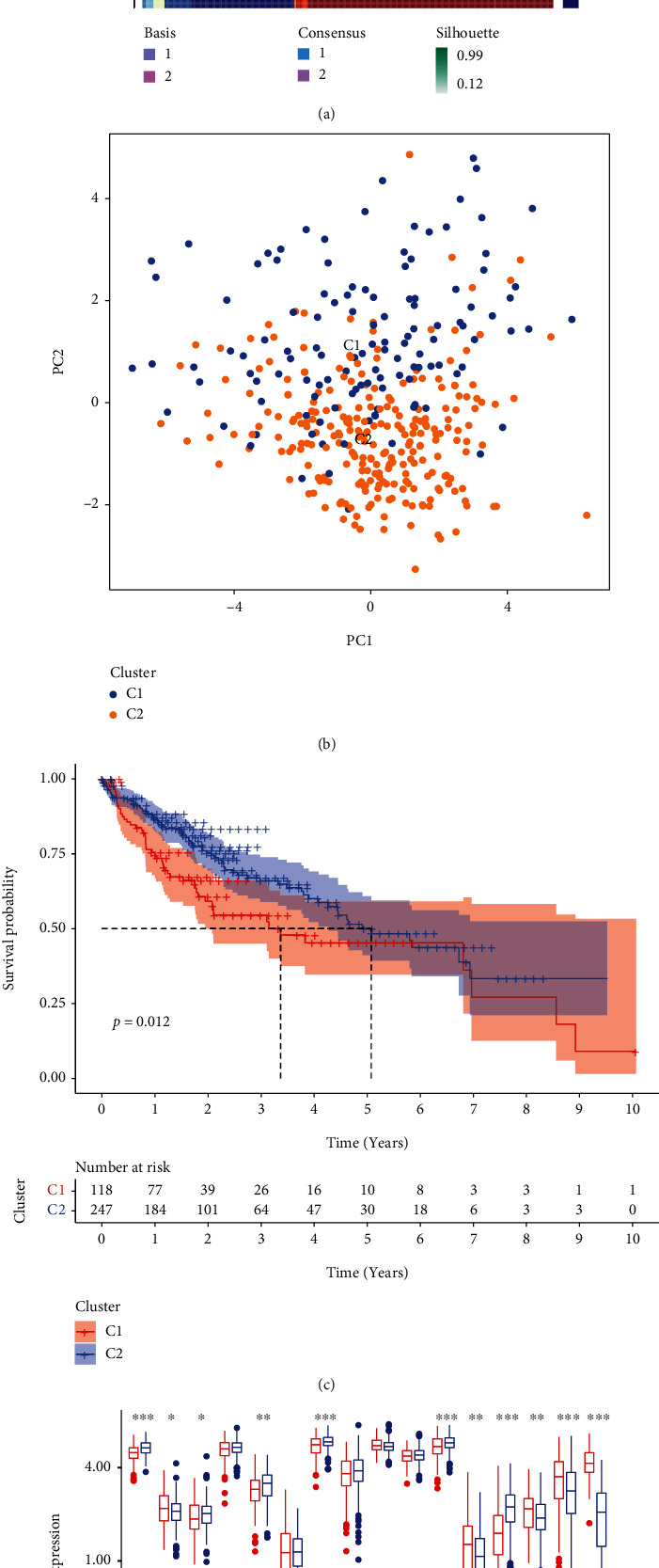
Construction and validation of the cuproptosis-related molecular subtype. (a) Consensus matrix of NMF analysis. (b) PCA analysis of the molecular subtype. (c) The K-M plot of the molecular subtype. (d) Differential expression of CRGs between molecular types. ^∗^represents *p* < 0.05, ^∗∗^represents *p* < 0.01, and ^∗∗∗^represents *p* < 0.001.

Two clusters were identified after NMF-clustering analysis (Figure 4(a) and Supplementary Figure 2). The PCA analysis shows that HCC samples are distinctly separated into two clusters (Figure 4(b)). The K-M analysis between the C1 and the C2 suggests that the overall survival of patients in C2 is better than in C1 (Figure 4(c)). CRGs including FDX1, LIAS, DBT, DLST, SLC31A1, and ATP7B are significantly higher expressed in C2, while ATP7A, MTF1, GLS, and CDKN2A are the opposite (Figure 4(d)). According to these findings, the two molecular subtypes behave differently in cuproptosis.

#### 3.2.2. Differentially Enriched GSVA Function between the Molecular Subtypes

**Figure 5:**
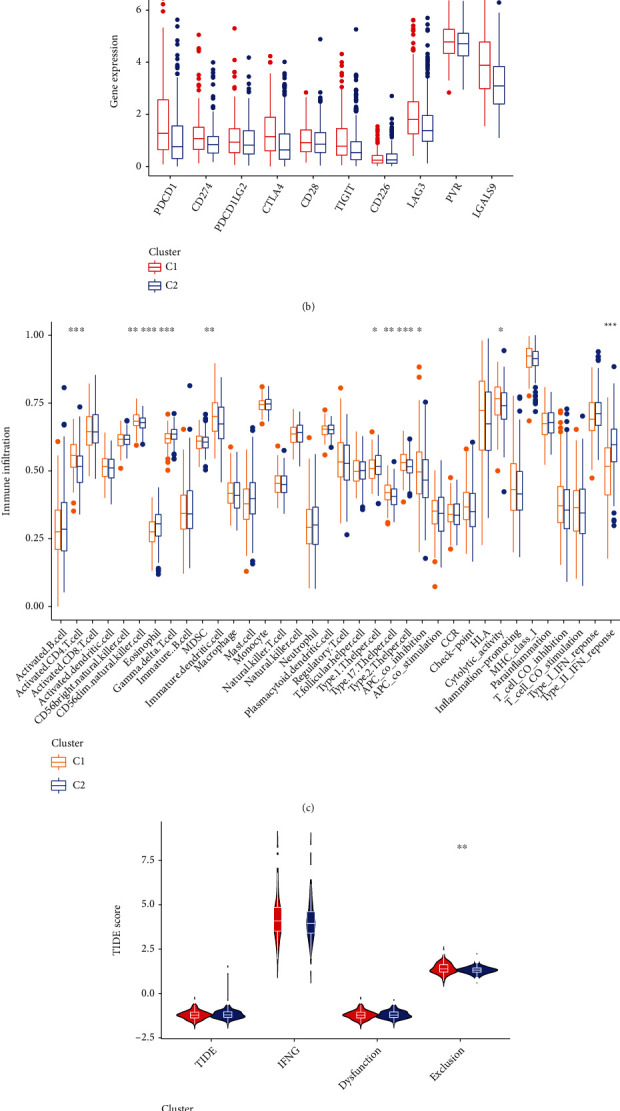
Function enrichment and immune landscape of the cuproptosis-related molecular subtype. (a) GSVA heat map of the molecular subtype. (b) Differential expression of immune checkpoint genes between molecular types. (c) ssGSEA analysis of the molecular subtype. (d) TIDE score of the molecular subtype. (e) Immune-stromal component evaluation of the molecular subtype. ^∗^ represents *p* < 0.05, ^∗∗^ represents *p* < 0.01, and ^∗∗∗^ represents *p* < 0.001.

The GSVA analysis shows the activation of KEGG pathways in C1/C2 HCC samples. Metabolism-related pathways such as fatty acid metabolism and drug metabolism, gene stability-related pathways such as DNA replication and mismatch repair, cell cycle, and citrate cycle tricarboxylic acid (TCA) cycle are significantly enriched (Figure 5(a) and Supplementary Table 1). The disorder of TCA cycle caused by excess copper directly result in the occurrence of cuproptosis, and the significantly enriched TCA cycle pathway indeed proves the rationale of the molecular subtype.

#### 3.2.3. Comparison of Immune Checkpoint Genes, Immune Cells, and Immune Score Levels between the Molecular Subtypes

Immune checkpoint genes including PDCD1, CD274, CTLA4, TIGIT, LAG3, and LGALS9 are significantly expressed lower in C2 (Figure 5(b)). The results of ssGSEA analysis show that the activated CD4^+^ T cell, CD56^dim^ natural killer cell, eosinophil, MDSC, type 2 T helper cell, type 1 T helper cell, and type 17 T helper cell are differentially infiltrated between the molecular subtypes (Figure 5(c)). A total of 3 kinds of immune response, including APC costimulation, HLA, and type II IFN response are differentially enriched between the subtypes (Figure 5(c)). The exclusion score is significantly lower in C2, while the dysfunction score and the integrated TIDE score do not present differences between molecular subgroups (Figure 5(d)). Moreover, the StromalScore of C2 is higher than C1, while the ImmuneScore is the opposite (Figure 5(e)). These results suggest that the tumor microenvironment driven by cuproptosis of the two molecular subtypes is diverse from each other.

### 3.3. Construction and Validation of CRG-Related Signature

#### 3.3.1. A CRG-Related Signature with Independent Prognostic Effect Was Constructed

**Figure 6:**
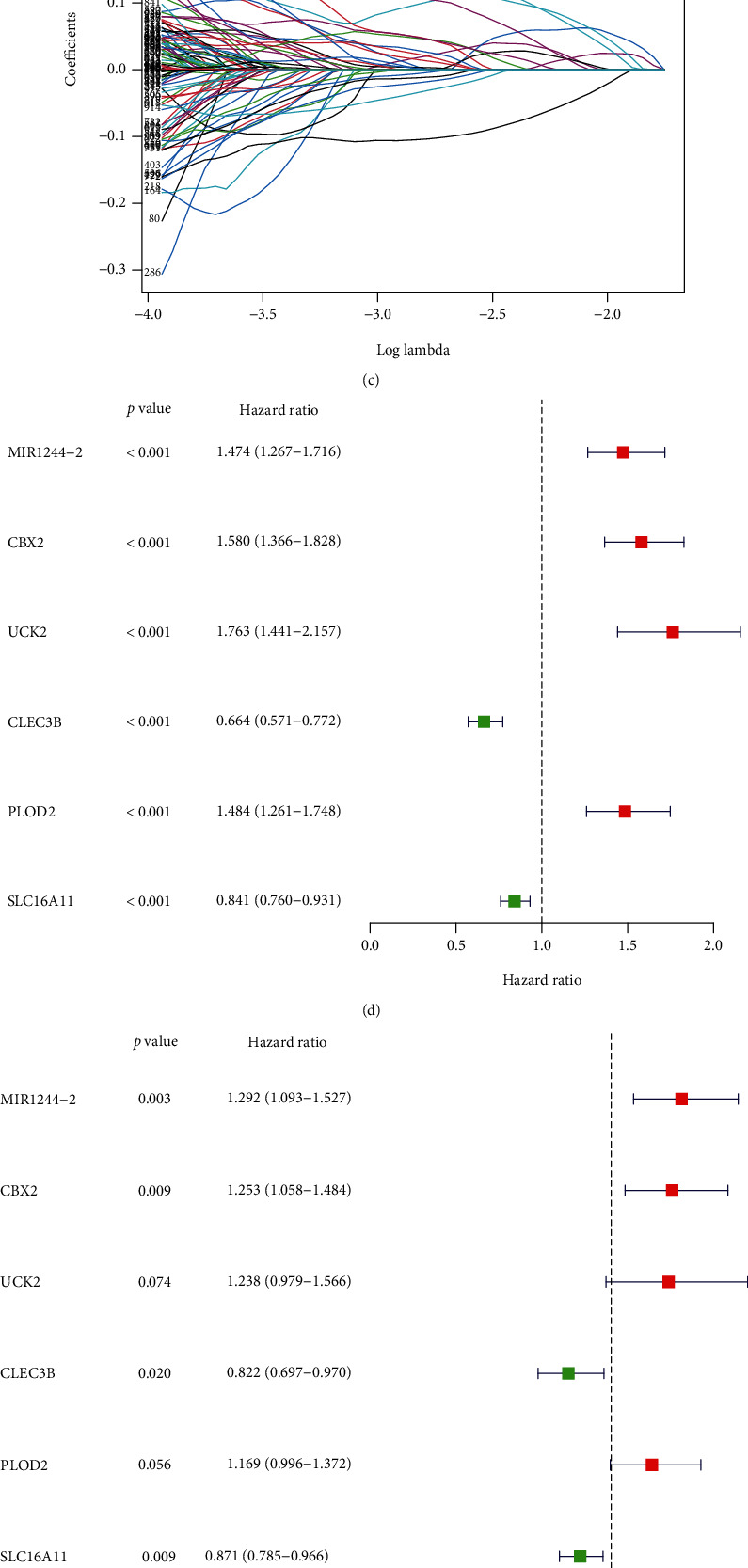
Construction of the cuproptosis-related prognostic signature using the TCGA-LIHC project. (a) Volcano map of the DEGs between molecular subtypes. (b) Distribution of partial likelihood deviation of the LASSO regression. (c) LASSO coefficient profiles. (d) Univariate Cox analysis of the 6 genes in the signature. (e) Multivariate Cox regression analysis of the 6 genes in the signature. (f) Cutoff point used to distinguish the low- and high-risk group. (g) ROC curve for predicting 1-, 2-, and 3-year overall survival. (h) ROC comparison between the signature and tumor stage, histologic grade, age, and gender.

A 6-gene signature was constructed based on the 1479 DEGs between molecular subtypes with univariate Cox regression analysis and LASSO regression analysis (Figures 6(a)–6(d)). Multivariate Cox regression analysis of the 6 genes shows that MIR1244-2, CBX2, CLEC3B, and SLC16A11 are independent prognostic factors (Figure 6(e)). The cutoff point was set as 1.570 according to the AIC (Figure 6(f)). The AUC of 1-, 2-, and 3-year is 0.775, 0.768, and 0.757, respectively (Figure 6(g)), and the 1-year ROC was further compared with age (AUC = 0.534), gender (AUC = 0.513), histologic grade (AUC = 0.505), and TNM stage (AUC = 0.664) (Figure 6(h)).

**Figure 7:**
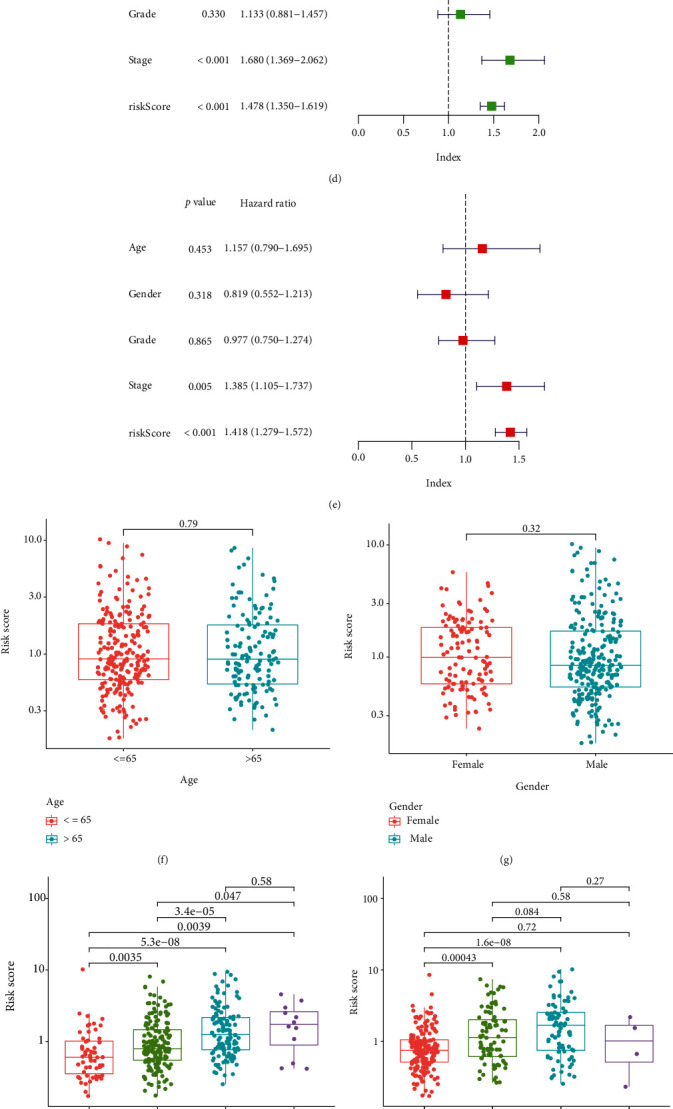
Validation of the cuproptosis-related prognostic signature in the TCGA-LIHC project. (a) The K-M plot of the signature. (b) Distribution of the survival states. (c) Distribution of the risk scores. (d) Univariate Cox analysis of the signature with clinical phenotypes. (e) Multivariate Cox regression analysis of the signature with clinical phenotypes. (f) Distribution of risk scores by age. (g) Distribution of risk scores by gender. (h) Distribution of risk scores by histologic grade. (i) Distribution of risk scores by tumor stage. (j) Differential expression of CRGs between the low- and the high-risk groups. (k) GSVA heat map of the signature. ^∗^represents p <0.05, ^∗∗^represents *p* < 0.01, and ^∗∗∗^represents *p* < 0.001.

HCC samples were divided into high- and low-risk groups in accordance with the AIC value. The overall survival of patients in low-risk group is superior to these in high-risk (Figures 7(a)–7(c)). The univariate Cox regression analysis indicates that the risk status (*p* < 0.001, HR = 1.478, and 95%CI [1.350–1.619]) and TNM stage (*p* < 0.001, HR = 1.680, and 95%CI [1.369–2.062]) are the risk factors for HCC patients, and the multivariate Cox regression analysis further proved that the risk score (*p* < 0.001, HR = 1.418, and 95%CI [1.279–1.572]) acts as an independent role (Figures 7(d) and 7(e)). There is no difference between age and gender in the distribution of risk scores, while increased risk scores are associated with worse histologic grade and more advanced TNM stage (Figures 7(f)–7(i)). CRGs including FDX1, DBT, GCSH, DLST, SLC31A1, and ATP7B are significantly highly expressed in the low-risk group, while LIPT1, DLAT, PDHA1, ATP7A, MTF1, GLS, and CDKN2A are the opposite (Figure 7(j)). Thus, the CRG-related signature represents an eligible AUC value as an independent prognostic factor and it is superior to other clinical phenotypes. The differential expression of CRGs between the two groups suggests that they perform differently on cuproptosis.

#### 3.3.2. Differentially Enriched GSVA Function between the Risk Groups

The results of GSVA analysis present the differentially enriched KEGG pathways in low-risk/high-risk HCC samples. Immune-related pathways such as T cell receptor signaling pathway and B cell receptor signaling pathway, gene stability-related pathways such as DNA replication and mismatch repair, cell cycle, and citrate cycle TCA cycle are significantly enriched (Figure 7(k) and Supplementary Table 2). It is apparent from these data that the signature inherits the molecular characteristics of the molecular subtype.

#### 3.3.3. Comparison of Immune Checkpoint Genes, Immune Cells, and Immune Score Levels between the Two Risk Groups

**Figure 8:**
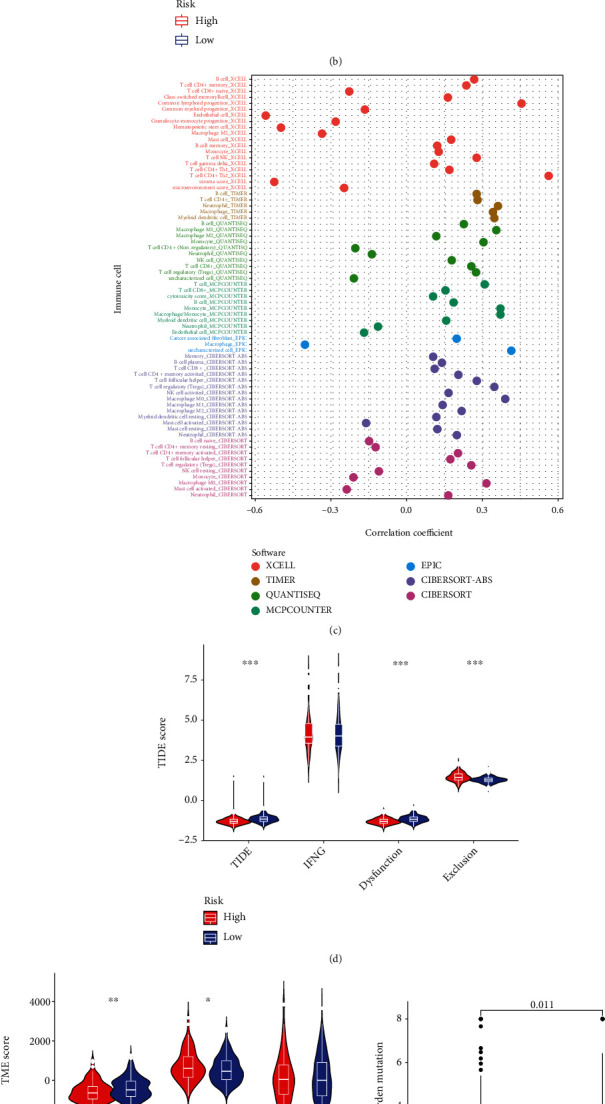
Immune landscape of the cuproptosis-related signature. (a) ssGSEA analysis of the signature. (b) Differential expression of immune checkpoint genes between the low- and high-risk groups. (c) The correlation of tumor-infiltrating immune cells and risk scores based on 7 algorithms. (d) TIDE score of the signature. (e) Immune-stromal component evaluation of the signature. (f) Comparison of TMB levels between the low- and high-risk groups. (g) The correlation between TMB level and risk score. ^∗^represents *p* < 0.05, ^∗∗^represents *p* < 0.01, and ^∗∗∗^represents *p* < 0.001.

The results of ssGSEA analysis show that the activated CD4^+^ T cell, activated dendritic cell, eosinophil, gamma-delta T cell, MDCS, regulatory T cell, type 1 T helper cell, and type 2 T helper cell are differentially infiltrated between the risk groups (Figure 8(a)). Immune functions including APC costimulation, HLA, MHC class I, type I IFN response, and type I IFN response are differentially enriched between the risk groups (Figure 8(a)). Immune checkpoint genes including PDCD1, CTLA4, TIGIT, LAG3, PVR, and LGALS9 are significantly expressed lower in the low-risk group (Figure 8(b)).

Correlations between risk scores and tumor-infiltrating immune cells are presented in Figure 8(c) and Supplementary Table 3. The risk score is strongly and negatively related to the infiltration level of endothelial cell, hematopoietic stem cell, macrophage M2, while positively correlated to T cell CD4+ Th2, neutrophil, macrophage, myeloid dendritic cell, macrophage M1, monocyte, T cell regulatory, and NK cell. The exclusion score is significantly higher in the low-risk group, while the dysfunction score and the integrated TIDE score are the opposite (Figure 8(d)). The StromalScore of the high-risk group was lower than the low-risk group, while ImmuneScore was the opposite (Figure 8(e)). Moreover, the risk score is positively related to the TMB score (Figures 8(f) and 8(g)). The tumor microenvironment driven by cuproptosis of the two risk groups differs from each other, which indicates that the signature can be used to predict the immune status of patients with HCC.

#### 3.3.4. Predicting the IC_50_ of Anti-HCC Agents

**Figure 9:**
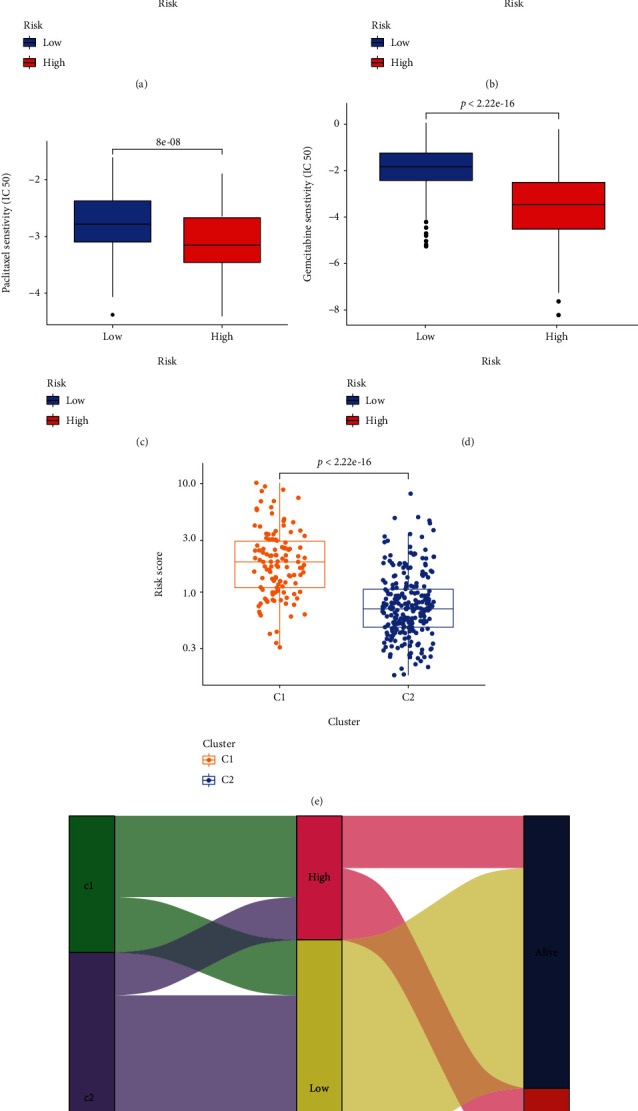
Drug-sensitive prediction and correlation between the cuproptosis-related molecular subtype and the cuproptosis-related signature. (a–d) Comparison of IC_50_ levels for sorafenib, cisplatin, paclitaxel, and gemcitabine between the low- and high-risk groups. (e) The distribution of risk scores between the molecular subtypes. (f) The Sankey diagram is used to present the distribution of molecular subtypes, risk status, and survival status.

Figures 9(a)–9(d) present the comparison of IC_50_ for sorafenib, cisplatin, paclitaxel, and gemcitabine between the low- and high-risk groups. The results show that HCC patients in the low-risk group have a higher IC_50_ in the four comparisons, which indicates that the patients in the high-risk group have a tendency to benefit more from the conventional agents. What stands out in this section is that the signature is able to recognize HCC patients who could benefit from conventional anti-HCC drugs.

#### 3.3.5. Correlations between Molecular Subtype and Signature

As shown in Figure 9(e), the risk score level in C1 is significantly higher than in C2. The distribution of patients by molecular subtype, risk score, and survival status are represented in Figure 9(f).

#### 3.3.6. The Signature Was Validated in the LIRI-JP and IMvigor210 Cohort

**Figure 10:**
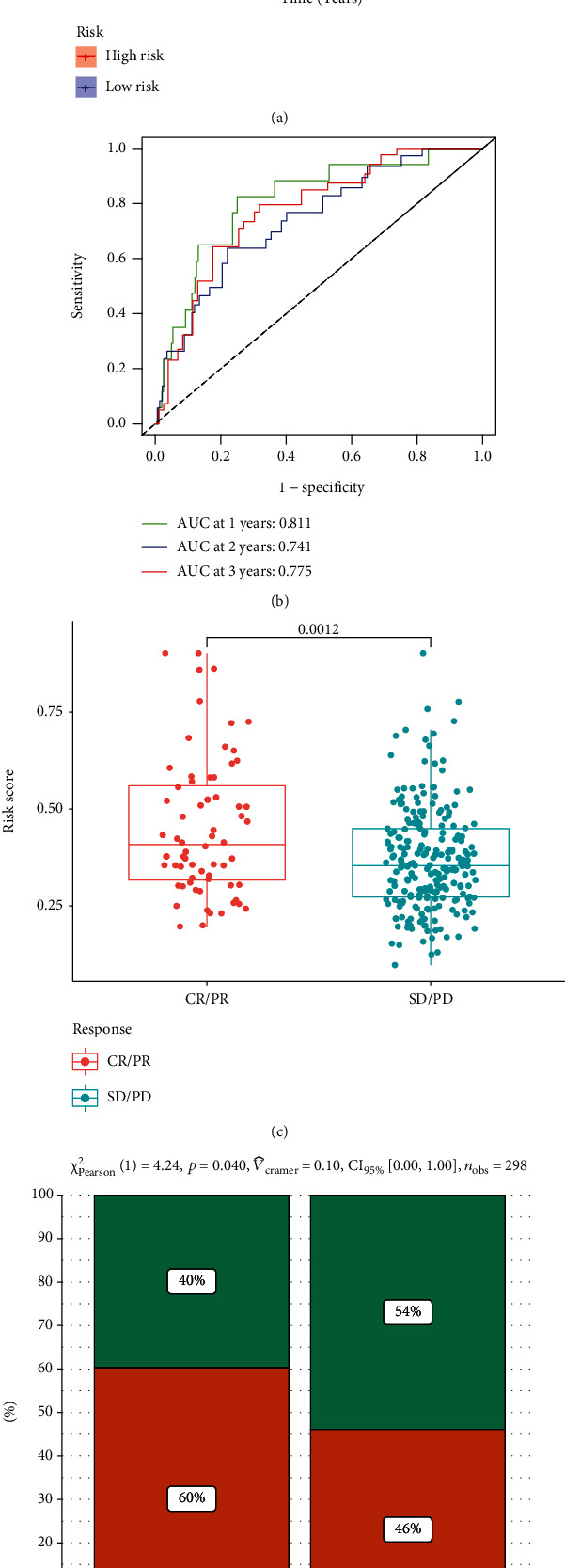
Validation of the signature in the LIRI-JP and IMvigor210 cohort. (a) K-M plot of the signature in the LIRI-JP cohort. (b) ROC curves for predicting 1-, 2-, and 3-year overall survival in the LIRI-JP cohort. (c and d) The ability of the signature to predict ICIs' response in the IMvigor210 cohort.

HCC patients in the LIRI-JP cohort were divided into low- and high-risk groups based on the previously constructed signature. The prognosis of patients in the low-risk group is superior to that of those in the high-risk group (Figure 10(a)). The AUC values of the 1-, 2-, and 3-year ROC curves are 0.811, 0.741, and 0.775, respectively (Figure 10(b)). Tumor patients who positively respond to ICIs have relatively higher risk scores, and tumor patients in the high-risk group are more likely to benefit from ICIs (Figures 10(c) and 10(d)). In summary, for the informants in this section, the signature is robust in predicting the prognosis of HCC and has the potential to foresee the response to immunotherapy.

## 4. Discussion

Cuproptosis was a novel form of regulated cell death proposed by Tsvetkov et al. [15]. It is revealed that the excess intracellular Cu disturbs the mitochondrial TCA cycle, leading to cell death and independent of other cell death pathways [31]. Although the growth inhibition of the elesclomol-Cu complex in HCC-related cell lines was not examined, it has been demonstrated that there is a strong association between Cu metabolism and HCC cell death. Nurmamat et al. synthesized a novel copper (II) complex, which could induce the cell cycle arrest and result in apoptosis in BEL-7404 cells [32]. Cu transporter-related genes such as ATP7A, ATP7B, and SLC31A1 were singularly expressed or copied in liver cancer samples and a relative higher level of Cu was observed in HCC cell [33]. Clinical studies have shown that the high levels of serum copper are associated with poor prognosis in patients with HCC (*p* < 0.01, HR = 2.06, and 95%CI [1.036–3.11]) [34]. What is more, Cu propelled the antitumor effects of troxerutin, a rutin derivative, in Huh-7 cells with little toxicity in normal cells [35]. Thus, an abundance of indirect evidence from laboratory to clinical has supported that Cu metabolism is strongly associated with HCC, and targeting CRGs is a potential therapeutic strategy for HCC. A comprehensive bioinformatics analysis of CRGs for molecular subtype identification and prognosis in HCC patients is important.

The molecular characteristic of CRGs in HCC was analyzed in this study. Most of the CRGs were differentially expressed between normal and tumor samples. FDX1 is the key regulator of cuproptosis with the function of inducing protein lipoylation when being deleted, which further results in resistance to cuproptosis [36]. FDX1 is shown to be a favorable factor for HCC patients in our study. Cu transporter-related genes including ATP7A, SLC31A1, and TCA-related genes including DBT, DLST, and DLAT are differentially expressed between normal and tumor samples. The expression of CRGs is associated with their CNV level, which indicates that CNV may contribute the CRGs heterogeneity in HCC. The CNV of Cu transporter-related genes is accompanied by a worse prognosis, which was anteriorly discussed by Davis et al. [33]. GCSH and PDHA1, both involved in protein lipoylation, are predicted to be associated with immunotherapy, however, their concrete role in immunotherapy has not been investigated.

Both molecular subtypes and signatures have significant differences in OS, with C2 superior to C1 and low-risk superior to high-risk. The molecular subtype and signature are strongly associated with cuproptosis, as most of the CRGs are differentially expressed between different molecular subtypes and risk groups. Highly expressed FDX1 and SLC31A1 are steadily associated with better prognosis in HCC patients, while ATP7A, MTF1, GLS, and CDKN2A are the opposite. MTF1, GLS, and CDKN2A were proved to be negative hits in the process of genome-wide CRISPR-Cas9 screen [15]. MTF1 was recently identified to be a propellant of tumor proliferation and metastasis in HCC cells and Hep3B-derived xenografts and was regulated by miR-148a-3p [37]. Knockdown or suppression of GLS impaired HCC cell proliferation and the expression level of GLS was modulated by nuclear factor-*κ*B [38]. The expression of CDKN2A is inversely correlated with cyclin D-CDK4/6-retinoblastoma protein, which was a potential biomarker for ribociclib in HCC [39]. Genomic loss or deletion of CDKN2A was identified to be a risk factor for ICI therapy [40].

Advanced HCC patients have a paucity of curative systemic therapy options. Even though, clinical trials have shown that HCC patients could benefit from ICIs. Atezolizumab, a monoclonal antibody targeting PD-L1, combined with bevacizumab, prolonged the median progression-free survival to 6.8 months versus 4.3 months in the sorafenib group [5]. Pembrolizumab is an anti-PD-1 monoclonal antibody and was approved for advanced HCC which progressed after sorafenib by FDA. The median OS observed in pembrolizumab cohort, a cohort that included HCC patients who progressed after sorafenib treatment, was 12.9 months [41]. Nivolumab is used in combination with ipilimumab with an ORR of 32% and a median OS of 22.8 months [42]. Tremelimumab is a monoclonal antibody that binds to CTLA-4. The median OS in a phase I/II randomized clinical study of the combination of tremelimumab and durvalumab for advanced HCC was 18.73 months [43]. The expression level of immune checkpoint genes is considered to be associated with the response to ICIs [44]. HCC patients in the C1 cluster and high-risk group may be positive for ICIs since most of the immune checkpoint genes are highly expressed in this population. Cu was identified to be a PD-L1 regulator in HCC, when combined with disulfiram, a blocker of enzyme acetaldehyde dehydrogenase, it could upregulate the expression of PD-L1 [45]. TMB is defined as the total number of mutations detected per million bases [46]. A higher TMB level means more expressed neoantigens on the cell surface, hence these patients who harbor high-TMB levels are sensitive to immunotherapy [47]. The risk score is significantly positively correlated with the TMB level in our signature.

Immune cell infiltration influences the prognosis of HCC and ICI response. Regulatory T cells, which are significantly enriched in the high-risk group, are able to suppress the expression of PD-L1, promote HCC progression, and correlate with poor survival [48]. The infiltration of macrophages was strongly positively associated with the risk scores. Macrophages are known as immunosuppressive cells in the tumor immune microenvironment and are associated with a worse prognosis [49]. It was reported that Cu depletion is a negative factor for immune system, exemplifying that the activity of macrophages is decreased in Cu depletion rat model [50]. Integrated TIDE score is developed to evaluate the response of ICIs, and a higher TIDE score is associated with worse ICI response [51]. Patients in the high-risk group have a lower level of integrated TIDE score and may be sensitive to ICIs. ESTIMATE is a tool for predicting tumor purity, as well as the presence of stromal cell and immune cell [52]. There are no significant differences in tumor purity between the molecular subtypes and risk groups.

The signature is tested as an independent factor for HCC patients, and the AUC value of the signature in predicting OS shows satisfactory predictive ability in the TCGA-LIHC project and LIRI-JP cohort. Previous studies have constructed signatures associated with other cell death formats based on the TCGA-LIHC project. Yang and Jiang constructed a necroptosis-related signature with necroptosis-related genes, and the 1- and 3-year AUC values were 0.741 and 0.648, respectively [53]. Wu et al. constructed a pyroptosis-related signature with pyroptosis-related genes, and the 1- and 3-year AUC values were 0.785 and 0.71 [54], respectively, while 0.760 and 0.708 in a pyroptosis-related long noncoding RNAs (lncRNAs) signature [55]. The 1- and 3-year ACU values in ferroptosis-related gene signature are 0.8 and 0.668 [56]. Wang et al. established an autophagy-related gene signature, and the 1- and 3-year AUC value was 0.781 [57]. A ferroptosis and pyroptosis molecular subtype-related signature has the AUC values of 0.805, 0.806, and 0.751 for 1-, 3-, and 5-year [16].

The signature has the potential to predict drug response for HCC patients. HCC patients in the high-risk group are more likely to benefit from conventional anti-HCC agents and ICI therapies.

## 5. Conclusion

In summary, we presented the molecular characterization of CRGs in HCC and constructed a prognostic signature for HCC based on cuproptosis-related molecular subtype. Both the molecular subtype and the signature are strongly associated with cuproptosis and HCC prognosis, as well as exhibiting an abundant immune landscape.

## Data Availability

The datasets used and/or analyzed in the current study are available in the article or supplementary material.

## Supplementary Materials

Supplementary 1 Supplementary Figure 1. Correlations between the CNV and gene expression of CRGs.

Supplementary 2 Supplementary Figure 2. Rank survey of the NMF analysis.

Supplementary 3 Supplementary Table 1. Detailed GSVA analysis of cuproptosis-related molecular subtype. Supplementary Table 2. Detailed GSVA analysis of cuproptosis-related signature. Supplementary Table 3. Detailed correlation analysis between risk scores and tumor-infiltrating immune cells.
