# Supplementary figures and images for: Cuproptosis-Related Signature Predicts the Prognosis, Tumor Microenvironment, and Drug Sensitivity of Hepatocellular Carcinoma
Source: J Immunol Res. 2022 Nov 16;2022:3393027. doi: 10.1155/2022/3393027 (PMC9691390; doi:10.1155/2022/3393027)

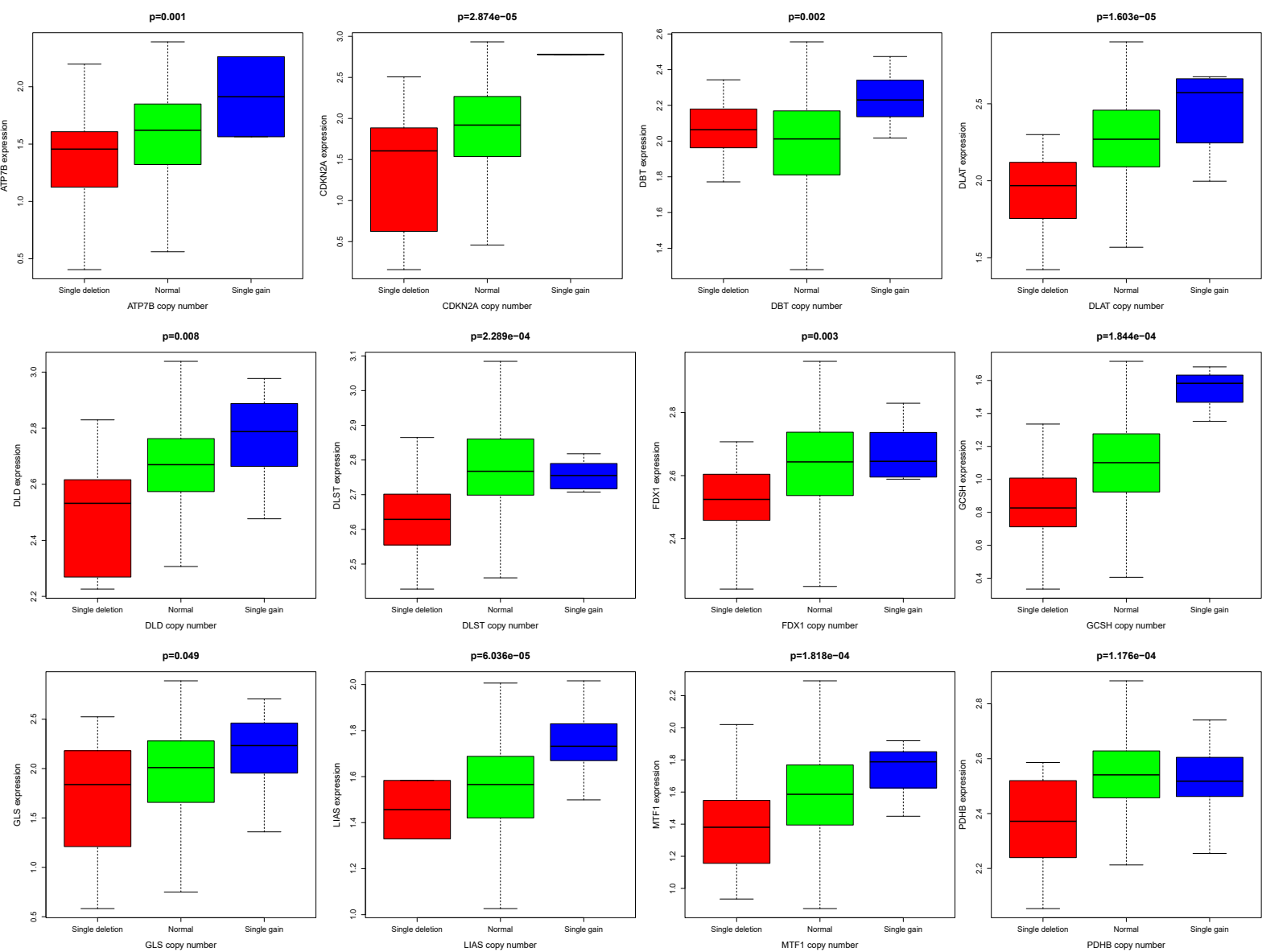

Supplement: Supplementary 1 — Supplementary Figure 1. Correlations between the CNV and gene expression of CRGs. [file 3393027.f1.pdf]

# NMF rank survey

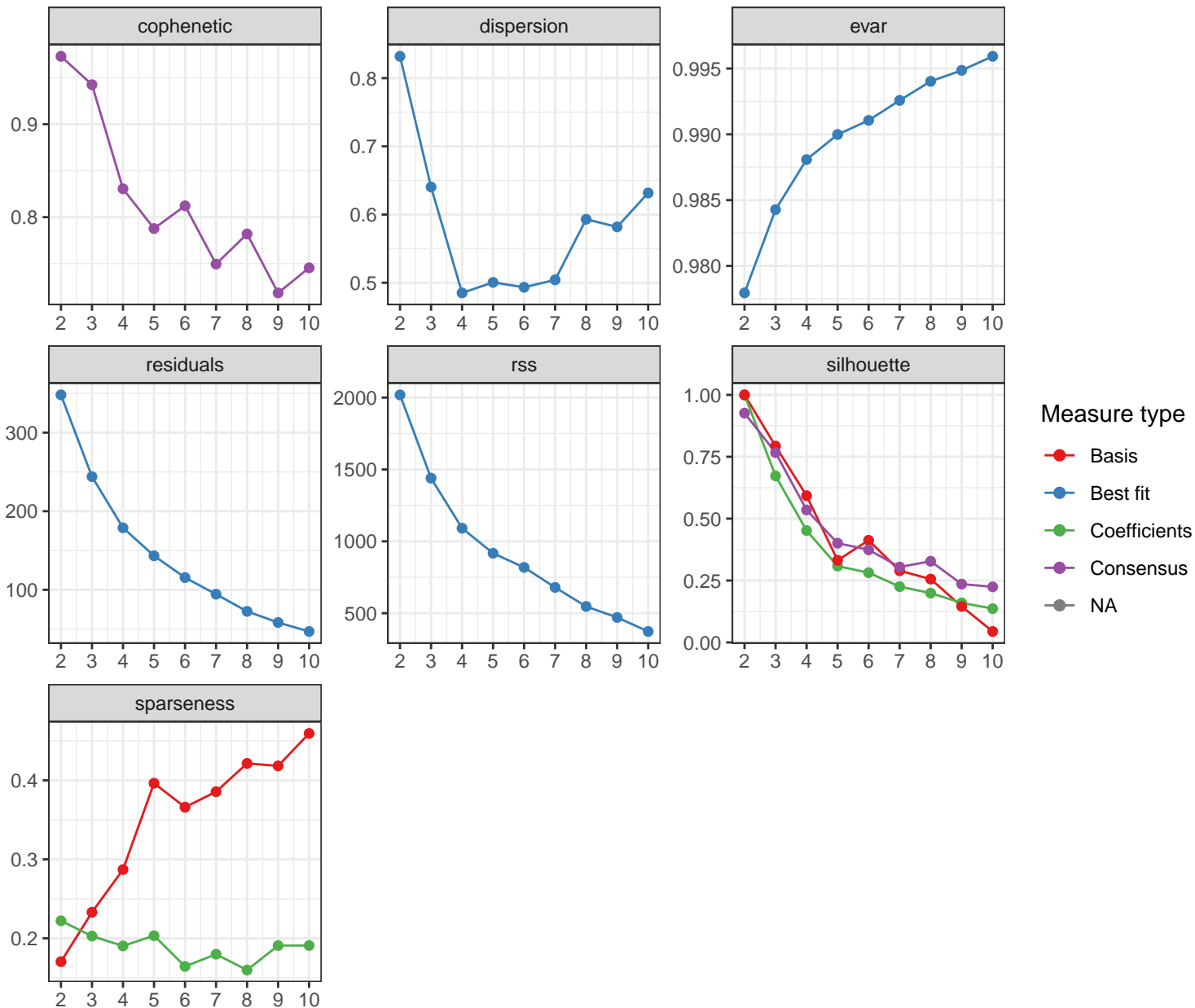

Factorization rank

Supplement: Supplementary 2 — Supplementary Figure 2. Rank survey of the NMF analysis. [file 3393027.f2.pdf]
