# Supplementary material for: Cuproptosis-Related Signature Predicts the Prognosis, Tumor Microenvironment, and Drug Sensitivity of Hepatocellular Carcinoma
Source: J Immunol Res. 2022 Nov 16;2022:3393027. doi: 10.1155/2022/3393027 (PMC9691390; doi:10.1155/2022/3393027)
Supplement: Supplementary 3 — Supplementary Table 1. Detailed GSVA analysis of cuproptosis-related molecular subtype. Supplementary Table 2. Detailed GSVA analysis of cuproptosis-related signature. Supplementary Table 3. Detailed correlation analysis between risk scores and tumor-infiltrating immune cells. [file 3393027.f3.docx]

**Supplementary Table 1.** Detailed GSVA analysis of cuproptosis-related molecular subtype.

| ID | P.Value | adj.P.Val |
| --- | --- | --- |
| KEGG_ARGININE_AND_PROLINE_METABOLISM | 6.14E-20 | 1.14E-17 |
| KEGG_PPAR_SIGNALING_PATHWAY | 2.09E-18 | 1.44E-16 |
| KEGG_ALANINE_ASPARTATE_AND_GLUTAMATE_METABOLISM | 2.32E-18 | 1.44E-16 |
| KEGG_PROPANOATE_METABOLISM | 4.04E-18 | 1.88E-16 |
| KEGG_DNA_REPLICATION | 6.96E-18 | 2.18E-16 |
| KEGG_FATTY_ACID_METABOLISM | 7.05E-18 | 2.18E-16 |
| KEGG_VALINE_LEUCINE_AND_ISOLEUCINE_DEGRADATION | 5.86E-17 | 1.56E-15 |
| KEGG_BETA_ALANINE_METABOLISM | 5.49E-16 | 1.28E-14 |
| KEGG_NITROGEN_METABOLISM | 1.12E-15 | 2.30E-14 |
| KEGG_HOMOLOGOUS_RECOMBINATION | 3.28E-15 | 6.10E-14 |
| KEGG_LINOLEIC_ACID_METABOLISM | 3.87E-15 | 6.54E-14 |
| KEGG_PROXIMAL_TUBULE_BICARBONATE_RECLAMATION | 4.95E-15 | 7.68E-14 |
| KEGG_TRYPTOPHAN_METABOLISM | 2.30E-14 | 3.29E-13 |
| KEGG_COMPLEMENT_AND_COAGULATION_CASCADES | 3.09E-14 | 4.11E-13 |
| KEGG_PRIMARY_BILE_ACID_BIOSYNTHESIS | 1.42E-13 | 1.76E-12 |
| KEGG_TYROSINE_METABOLISM | 2.20E-13 | 2.56E-12 |
| KEGG_BUTANOATE_METABOLISM | 4.38E-13 | 4.79E-12 |
| KEGG_CELL_CYCLE | 7.65E-13 | 7.90E-12 |
| KEGG_LIMONENE_AND_PINENE_DEGRADATION | 1.50E-12 | 1.47E-11 |
| KEGG_DRUG_METABOLISM_CYTOCHROME_P450 | 2.80E-12 | 2.60E-11 |
| KEGG_PHENYLALANINE_METABOLISM | 3.89E-12 | 3.38E-11 |
| KEGG_PEROXISOME | 4.00E-12 | 3.38E-11 |
| KEGG_CITRATE_CYCLE_TCA_CYCLE | 4.50E-11 | 3.64E-10 |
| KEGG_BASE_EXCISION_REPAIR | 4.72E-11 | 3.66E-10 |
| KEGG_RETINOL_METABOLISM | 7.58E-11 | 5.64E-10 |
| KEGG_HISTIDINE_METABOLISM | 2.13E-10 | 1.53E-09 |
| KEGG_PYRUVATE_METABOLISM | 3.09E-10 | 2.13E-09 |
| KEGG_GLYCINE_SERINE_AND_THREONINE_METABOLISM | 3.77E-10 | 2.51E-09 |
| KEGG_RENIN_ANGIOTENSIN_SYSTEM | 4.53E-10 | 2.91E-09 |
| KEGG_STEROID_HORMONE_BIOSYNTHESIS | 1.17E-09 | 7.24E-09 |
| KEGG_BIOSYNTHESIS_OF_UNSATURATED_FATTY_ACIDS | 1.67E-09 | 1.00E-08 |
| KEGG_MISMATCH_REPAIR | 3.88E-09 | 2.25E-08 |
| KEGG_ALPHA_LINOLENIC_ACID_METABOLISM | 5.03E-09 | 2.84E-08 |
| KEGG_METABOLISM_OF_XENOBIOTICS_BY_CYTOCHROME_P450 | 7.88E-09 | 4.31E-08 |
| KEGG_SPLICEOSOME | 1.37E-08 | 7.30E-08 |
| KEGG_GLYCOLYSIS_GLUCONEOGENESIS | 3.38E-08 | 1.75E-07 |
| KEGG_PYRIMIDINE_METABOLISM | 2.89E-07 | 1.45E-06 |
| KEGG_GLYCEROLIPID_METABOLISM | 3.20E-07 | 1.57E-06 |
| KEGG_GLYOXYLATE_AND_DICARBOXYLATE_METABOLISM | 8.63E-07 | 4.12E-06 |
| KEGG_P53_SIGNALING_PATHWAY | 1.24E-06 | 5.78E-06 |
| KEGG_ADIPOCYTOKINE_SIGNALING_PATHWAY | 1.37E-06 | 6.23E-06 |
| KEGG_TAURINE_AND_HYPOTAURINE_METABOLISM | 1.49E-06 | 6.57E-06 |
| KEGG_NUCLEOTIDE_EXCISION_REPAIR | 1.52E-06 | 6.57E-06 |
| KEGG_DRUG_METABOLISM_OTHER_ENZYMES | 1.63E-06 | 6.90E-06 |
| KEGG_ASCORBATE_AND_ALDARATE_METABOLISM | 1.91E-06 | 7.91E-06 |
| KEGG_ARACHIDONIC_ACID_METABOLISM | 2.09E-06 | 8.46E-06 |
| KEGG_LYSINE_DEGRADATION | 2.84E-06 | 1.13E-05 |
| KEGG_MATURITY_ONSET_DIABETES_OF_THE_YOUNG | 8.56E-06 | 3.32E-05 |
| KEGG_OOCYTE_MEIOSIS | 9.26E-06 | 3.51E-05 |
| KEGG_ABC_TRANSPORTERS | 1.03E-05 | 3.84E-05 |
| KEGG_RNA_POLYMERASE | 1.38E-05 | 5.05E-05 |
| KEGG_PATHOGENIC_ESCHERICHIA_COLI_INFECTION | 2.21E-05 | 7.91E-05 |
| KEGG_STARCH_AND_SUCROSE_METABOLISM | 2.75E-05 | 9.66E-05 |
| KEGG_PORPHYRIN_AND_CHLOROPHYLL_METABOLISM | 3.73E-05 | 0.000128 |
| KEGG_PENTOSE_AND_GLUCURONATE_INTERCONVERSIONS | 4.64E-05 | 0.000157 |
| KEGG_BLADDER_CANCER | 9.56E-05 | 0.000317 |
| KEGG_PROTEASOME | 0.000332 | 0.001028 |
| KEGG_RNA_DEGRADATION | 0.000432 | 0.001317 |
| KEGG_TERPENOID_BACKBONE_BIOSYNTHESIS | 0.000485 | 0.001432 |
| KEGG_STEROID_BIOSYNTHESIS | 0.00061 | 0.001772 |
| KEGG_RIBOSOME | 0.001235 | 0.003479 |
| KEGG_NON_HOMOLOGOUS_END_JOINING | 0.015804 | 0.03721 |
| KEGG_PRIMARY_IMMUNODEFICIENCY | 0.02061 | 0.045635 |

**Supplementary Table 2.** Detailed GSVA analysis of cuproptosis-related signature.

| ID | P.Value | adj.P.Val |
| --- | --- | --- |
| KEGG_PRIMARY_BILE_ACID_BIOSYNTHESIS | 2.69E-31 | 5.01E-29 |
| KEGG_FATTY_ACID_METABOLISM | 1.19E-28 | 1.10E-26 |
| KEGG_PPAR_SIGNALING_PATHWAY | 1.47E-27 | 9.12E-26 |
| KEGG_GLYCINE_SERINE_AND_THREONINE_METABOLISM | 4.12E-27 | 1.92E-25 |
| KEGG_DRUG_METABOLISM_CYTOCHROME_P450 | 5.66E-27 | 2.10E-25 |
| KEGG_TYROSINE_METABOLISM | 1.81E-26 | 5.61E-25 |
| KEGG_RETINOL_METABOLISM | 3.35E-25 | 8.91E-24 |
| KEGG_ARGININE_AND_PROLINE_METABOLISM | 3.88E-25 | 9.03E-24 |
| KEGG_BETA_ALANINE_METABOLISM | 6.94E-25 | 1.43E-23 |
| KEGG_BUTANOATE_METABOLISM | 8.32E-25 | 1.55E-23 |
| KEGG_TRYPTOPHAN_METABOLISM | 1.71E-24 | 2.88E-23 |
| KEGG_HISTIDINE_METABOLISM | 5.40E-24 | 8.37E-23 |
| KEGG_LINOLEIC_ACID_METABOLISM | 2.38E-23 | 3.40E-22 |
| KEGG_PHENYLALANINE_METABOLISM | 4.30E-23 | 5.71E-22 |
| KEGG_ALANINE_ASPARTATE_AND_GLUTAMATE_METABOLISM | 6.20E-23 | 7.68E-22 |
| KEGG_STEROID_HORMONE_BIOSYNTHESIS | 1.23E-22 | 1.43E-21 |
| KEGG_PEROXISOME | 1.93E-22 | 2.11E-21 |
| KEGG_VALINE_LEUCINE_AND_ISOLEUCINE_DEGRADATION | 2.51E-22 | 2.60E-21 |
| KEGG_METABOLISM_OF_XENOBIOTICS_BY_CYTOCHROME_P450 | 3.93E-22 | 3.85E-21 |
| KEGG_PROPANOATE_METABOLISM | 1.62E-21 | 1.51E-20 |
| KEGG_PROXIMAL_TUBULE_BICARBONATE_RECLAMATION | 1.96E-21 | 1.74E-20 |
| KEGG_LIMONENE_AND_PINENE_DEGRADATION | 3.37E-21 | 2.85E-20 |
| KEGG_COMPLEMENT_AND_COAGULATION_CASCADES | 2.25E-20 | 1.82E-19 |
| KEGG_NITROGEN_METABOLISM | 2.17E-19 | 1.68E-18 |
| KEGG_CELL_CYCLE | 2.01E-18 | 1.45E-17 |
| KEGG_DNA_REPLICATION | 2.08E-18 | 1.45E-17 |
| KEGG_SPLICEOSOME | 2.10E-18 | 1.45E-17 |
| KEGG_DRUG_METABOLISM_OTHER_ENZYMES | 5.69E-18 | 3.78E-17 |
| KEGG_HOMOLOGOUS_RECOMBINATION | 3.19E-17 | 2.04E-16 |
| KEGG_PYRUVATE_METABOLISM | 2.17E-16 | 1.34E-15 |
| KEGG_PATHOGENIC_ESCHERICHIA_COLI_INFECTION | 2.66E-16 | 1.59E-15 |
| KEGG_RNA_POLYMERASE | 6.67E-15 | 3.88E-14 |
| KEGG_STARCH_AND_SUCROSE_METABOLISM | 1.22E-14 | 6.87E-14 |
| KEGG_ASCORBATE_AND_ALDARATE_METABOLISM | 3.37E-14 | 1.84E-13 |
| KEGG_GLYCOLYSIS_GLUCONEOGENESIS | 6.61E-14 | 3.51E-13 |
| KEGG_ARACHIDONIC_ACID_METABOLISM | 7.63E-13 | 3.94E-12 |
| KEGG_BIOSYNTHESIS_OF_UNSATURATED_FATTY_ACIDS | 8.62E-13 | 4.33E-12 |
| KEGG_NON_HOMOLOGOUS_END_JOINING | 1.15E-12 | 5.64E-12 |
| KEGG_RNA_DEGRADATION | 1.61E-12 | 7.68E-12 |
| KEGG_GLYOXYLATE_AND_DICARBOXYLATE_METABOLISM | 4.61E-12 | 2.14E-11 |
| KEGG_MISMATCH_REPAIR | 5.08E-12 | 2.30E-11 |
| KEGG_RENIN_ANGIOTENSIN_SYSTEM | 5.27E-12 | 2.34E-11 |
| KEGG_PYRIMIDINE_METABOLISM | 1.88E-11 | 8.11E-11 |
| KEGG_BASE_EXCISION_REPAIR | 2.13E-11 | 9.02E-11 |
| KEGG_PENTOSE_AND_GLUCURONATE_INTERCONVERSIONS | 2.31E-11 | 9.56E-11 |
| KEGG_ALPHA_LINOLENIC_ACID_METABOLISM | 3.03E-11 | 1.23E-10 |
| KEGG_ABC_TRANSPORTERS | 3.40E-11 | 1.35E-10 |
| KEGG_NUCLEOTIDE_EXCISION_REPAIR | 4.88E-11 | 1.89E-10 |
| KEGG_OOCYTE_MEIOSIS | 1.29E-10 | 4.88E-10 |
| KEGG_GLYCOSAMINOGLYCAN_BIOSYNTHESIS_KERATAN_SULFATE | 1.44E-10 | 5.36E-10 |
| KEGG_VIBRIO_CHOLERAE_INFECTION | 2.00E-10 | 7.30E-10 |
| KEGG_GLYCEROLIPID_METABOLISM | 2.06E-10 | 7.35E-10 |
| KEGG_PURINE_METABOLISM | 6.23E-10 | 2.19E-09 |
| KEGG_BLADDER_CANCER | 1.45E-09 | 5.00E-09 |
| KEGG_UBIQUITIN_MEDIATED_PROTEOLYSIS | 2.01E-09 | 6.79E-09 |
| KEGG_CITRATE_CYCLE_TCA_CYCLE | 4.27E-09 | 1.42E-08 |
| KEGG_TAURINE_AND_HYPOTAURINE_METABOLISM | 4.41E-09 | 1.44E-08 |
| KEGG_LYSINE_DEGRADATION | 4.68E-09 | 1.50E-08 |
| KEGG_ADIPOCYTOKINE_SIGNALING_PATHWAY | 6.41E-09 | 2.02E-08 |
| KEGG_P53_SIGNALING_PATHWAY | 7.56E-09 | 2.34E-08 |
| KEGG_AMINOACYL_TRNA_BIOSYNTHESIS | 1.17E-08 | 3.58E-08 |
| KEGG_FC_GAMMA_R_MEDIATED_PHAGOCYTOSIS | 2.21E-08 | 6.62E-08 |
| KEGG_GLYCOSPHINGOLIPID_BIOSYNTHESIS_LACTO_AND_NEOLACTO_SERIES | 8.18E-08 | 2.42E-07 |
| KEGG_PROTEASOME | 8.70E-08 | 2.53E-07 |
| KEGG_MATURITY_ONSET_DIABETES_OF_THE_YOUNG | 9.70E-08 | 2.78E-07 |
| KEGG_PORPHYRIN_AND_CHLOROPHYLL_METABOLISM | 1.03E-07 | 2.92E-07 |
| KEGG_FOLATE_BIOSYNTHESIS | 1.61E-07 | 4.47E-07 |
| KEGG_RIBOSOME | 1.71E-07 | 4.69E-07 |
| KEGG_CYTOSOLIC_DNA_SENSING_PATHWAY | 1.85E-07 | 5.00E-07 |
| KEGG_RIBOFLAVIN_METABOLISM | 1.96E-07 | 5.21E-07 |
| KEGG_SNARE_INTERACTIONS_IN_VESICULAR_TRANSPORT | 2.02E-07 | 5.28E-07 |
| KEGG_PROGESTERONE_MEDIATED_OOCYTE_MATURATION | 2.05E-07 | 5.30E-07 |
| KEGG_OLFACTORY_TRANSDUCTION | 4.85E-07 | 1.24E-06 |
| KEGG_CYSTEINE_AND_METHIONINE_METABOLISM | 6.31E-07 | 1.59E-06 |
| KEGG_VASOPRESSIN_REGULATED_WATER_REABSORPTION | 8.03E-07 | 1.99E-06 |
| KEGG_ENDOCYTOSIS | 1.99E-06 | 4.87E-06 |
| KEGG_BASAL_TRANSCRIPTION_FACTORS | 2.02E-06 | 4.87E-06 |
| KEGG_NOD_LIKE_RECEPTOR_SIGNALING_PATHWAY | 3.38E-06 | 8.00E-06 |
| KEGG_N_GLYCAN_BIOSYNTHESIS | 3.40E-06 | 8.00E-06 |
| KEGG_VALINE_LEUCINE_AND_ISOLEUCINE_BIOSYNTHESIS | 4.66E-06 | 1.08E-05 |
| KEGG_NICOTINATE_AND_NICOTINAMIDE_METABOLISM | 4.71E-06 | 1.08E-05 |
| KEGG_RENAL_CELL_CARCINOMA | 1.36E-05 | 3.08E-05 |
| KEGG_PROTEIN_EXPORT | 1.39E-05 | 3.12E-05 |
| KEGG_LYSOSOME | 2.18E-05 | 4.82E-05 |
| KEGG_EPITHELIAL_CELL_SIGNALING_IN_HELICOBACTER_PYLORI_INFECTION | 2.30E-05 | 5.04E-05 |
| KEGG_GLYCOSYLPHOSPHATIDYLINOSITOL_GPI_ANCHOR_BIOSYNTHESIS | 3.72E-05 | 8.04E-05 |
| KEGG_GLYCOSPHINGOLIPID_BIOSYNTHESIS_GLOBO_SERIES | 3.95E-05 | 8.44E-05 |
| KEGG_NOTCH_SIGNALING_PATHWAY | 4.35E-05 | 9.18E-05 |
| KEGG_PANCREATIC_CANCER | 5.08E-05 | 0.000106 |
| KEGG_THYROID_CANCER | 6.87E-05 | 0.000142 |
| KEGG_MTOR_SIGNALING_PATHWAY | 6.96E-05 | 0.000142 |
| KEGG_ERBB_SIGNALING_PATHWAY | 8.24E-05 | 0.000166 |
| KEGG_HUNTINGTONS_DISEASE | 9.85E-05 | 0.000197 |
| KEGG_NEUROTROPHIN_SIGNALING_PATHWAY | 0.000145 | 0.000287 |
| KEGG_RIG_I_LIKE_RECEPTOR_SIGNALING_PATHWAY | 0.000178 | 0.000348 |
| KEGG_GLYCOSPHINGOLIPID_BIOSYNTHESIS_GANGLIO_SERIES | 0.000284 | 0.000549 |
| KEGG_REGULATION_OF_ACTIN_CYTOSKELETON | 0.000289 | 0.000549 |
| KEGG_LEISHMANIA_INFECTION | 0.000289 | 0.000549 |
| KEGG_CHRONIC_MYELOID_LEUKEMIA | 0.00037 | 0.000696 |
| KEGG_T_CELL_RECEPTOR_SIGNALING_PATHWAY | 0.000457 | 0.00085 |
| KEGG_PATHWAYS_IN_CANCER | 0.000789 | 0.001453 |
| KEGG_NON_SMALL_CELL_LUNG_CANCER | 0.000901 | 0.001643 |
| KEGG_STEROID_BIOSYNTHESIS | 0.001015 | 0.001832 |
| KEGG_COLORECTAL_CANCER | 0.001031 | 0.001845 |
| KEGG_INOSITOL_PHOSPHATE_METABOLISM | 0.001185 | 0.0021 |
| KEGG_PHOSPHATIDYLINOSITOL_SIGNALING_SYSTEM | 0.001614 | 0.002806 |
| KEGG_ADHERENS_JUNCTION | 0.001803 | 0.003105 |
| KEGG_SMALL_CELL_LUNG_CANCER | 0.002213 | 0.003777 |
| KEGG_GLYCOSAMINOGLYCAN_BIOSYNTHESIS_CHONDROITIN_SULFATE | 0.003399 | 0.005696 |
| KEGG_TERPENOID_BACKBONE_BIOSYNTHESIS | 0.004582 | 0.007347 |
| KEGG_ACUTE_MYELOID_LEUKEMIA | 0.0054 | 0.008441 |
| KEGG_B_CELL_RECEPTOR_SIGNALING_PATHWAY | 0.006253 | 0.009533 |
| KEGG_PRIMARY_IMMUNODEFICIENCY | 0.013429 | 0.019514 |

**Supplementary Table 3.** Detailed correlation analysis between risk scores and tumor-infiltrating immune cells.

| Immune cell | Cor | P.Value |
| --- | --- | --- |
| B cell_TIMER | 0.277255 | 8.23E-08 |
| T cell CD4+_TIMER | 0.280665 | 5.63E-08 |
| Neutrophil_TIMER | 0.361653 | 1.40E-12 |
| Macrophage_TIMER | 0.341878 | 1.91E-11 |
| Myeloid dendritic cell_TIMER | 0.347431 | 1.16E-11 |
| B cell naive_CIBERSORT | -0.14875 | 0.0044 |
| T cell CD4+ memory resting_CIBERSORT | -0.12299 | 0.018741 |
| T cell CD4+ memory activated_CIBERSORT | 0.203293 | 9.17E-05 |
| T cell follicular helper_CIBERSORT | 0.172988 | 0.000905 |
| T cell regulatory (Tregs)_CIBERSORT | 0.255978 | 7.18E-07 |
| NK cell resting_CIBERSORT | -0.11008 | 0.035524 |
| Monocyte_CIBERSORT | -0.2109 | 4.88E-05 |
| Macrophage M0_CIBERSORT | 0.316031 | 6.57E-10 |
| Mast cell activated_CIBERSORT | -0.23742 | 4.52E-06 |
| Neutrophil_CIBERSORT | 0.16479 | 0.001582 |
| B cell memory_CIBERSORT-ABS | 0.104854 | 0.045297 |
| B cell plasma_CIBERSORT-ABS | 0.139618 | 0.007555 |
| T cell CD8+_CIBERSORT-ABS | 0.110708 | 0.034488 |
| T cell CD4+ memory activated_CIBERSORT-ABS | 0.204876 | 8.06E-05 |
| T cell follicular helper_CIBERSORT-ABS | 0.277715 | 6.89E-08 |
| T cell regulatory (Tregs)_CIBERSORT-ABS | 0.347332 | 8.69E-12 |
| NK cell activated_CIBERSORT-ABS | 0.165968 | 0.001462 |
| Macrophage M0_CIBERSORT-ABS | 0.390453 | 9.67E-15 |
| Macrophage M1_CIBERSORT-ABS | 0.142447 | 0.00641 |
| Macrophage M2_CIBERSORT-ABS | 0.21778 | 2.83E-05 |
| Myeloid dendritic cell resting_CIBERSORT-ABS | 0.117516 | 0.024753 |
| Mast cell activated_CIBERSORT-ABS | -0.16074 | 0.002067 |
| Mast cell resting_CIBERSORT-ABS | 0.121574 | 0.020162 |
| Neutrophil_CIBERSORT-ABS | 0.19887 | 0.000131 |
| B cell_QUANTISEQ | 0.226491 | 1.25E-05 |
| Macrophage M1_QUANTISEQ | 0.355046 | 2.77E-12 |
| Macrophage M2_QUANTISEQ | 0.117249 | 0.025085 |
| Monocyte_QUANTISEQ | 0.304446 | 2.88E-09 |
| Neutrophil_QUANTISEQ | -0.20345 | 9.05E-05 |
| NK cell_QUANTISEQ | -0.13786 | 0.0084 |
| T cell CD4+ (non-regulatory)_QUANTISEQ | 0.178537 | 0.000611 |
| T cell CD8+_QUANTISEQ | 0.25609 | 7.10E-07 |
| T cell regulatory (Tregs)_QUANTISEQ | 0.274867 | 9.48E-08 |
| uncharacterized cell_QUANTISEQ | -0.20916 | 5.87E-05 |
| T cell_MCPCOUNTER | 0.309086 | 1.96E-09 |
| T cell CD8+_MCPCOUNTER | 0.153807 | 0.00322 |
| cytotoxicity score_MCPCOUNTER | 0.104682 | 0.045691 |
| B cell_MCPCOUNTER | 0.186027 | 0.000361 |
| Monocyte_MCPCOUNTER | 0.371387 | 3.00E-13 |
| Macrophage/Monocyte_MCPCOUNTER | 0.371387 | 3.00E-13 |
| Myeloid dendritic cell_MCPCOUNTER | 0.156613 | 0.002723 |
| Neutrophil_MCPCOUNTER | -0.11366 | 0.029981 |
| Endothelial cell_MCPCOUNTER | -0.16869 | 0.001233 |
| B cell_XCELL | 0.267564 | 2.11E-07 |
| T cell CD4+ memory_XCELL | 0.236354 | 5.00E-06 |
| T cell CD8+ naive_XCELL | -0.22738 | 1.15E-05 |
| Class-switched memory B cell_XCELL | 0.163146 | 0.001765 |
| Common lymphoid progenitor_XCELL | 0.455092 | 4.64E-20 |
| Common myeloid progenitor_XCELL | -0.16547 | 0.001512 |
| Endothelial cell_XCELL | -0.5584 | 2.61E-31 |
| Granulocyte-monocyte progenitor_XCELL | -0.2813 | 4.59E-08 |
| Hematopoietic stem cell_XCELL | -0.49786 | 2.94E-24 |
| Macrophage M2_XCELL | -0.3353 | 4.86E-11 |
| Mast cell_XCELL | 0.176324 | 0.000715 |
| B cell memory_XCELL | 0.121031 | 0.02073 |
| Monocyte_XCELL | 0.126749 | 0.015393 |
| T cell NK_XCELL | 0.277158 | 7.34E-08 |
| T cell gamma delta_XCELL | 0.108842 | 0.037667 |
| T cell CD4+ Th1_XCELL | 0.16938 | 0.001161 |
| T cell CD4+ Th2_XCELL | 0.562387 | 7.94E-32 |
| stroma score_XCELL | -0.52439 | 0 |
| microenvironment score_XCELL | -0.24762 | 1.81E-06 |
| Cancer associated fibroblast_EPIC | 0.19808 | 0.000144 |
| Macrophage_EPIC | -0.40288 | 1.54E-17 |
| uncharacterized cell_EPIC | 0.414475 | 0 |
